# Effects of Intermittent Hypoxia and Electrical Muscle Stimulation on Cognitive and Physiological Metrics

**DOI:** 10.3390/bioengineering10050536

**Published:** 2023-04-27

**Authors:** Elizaveta Reganova, Ksenia Solovyeva, Dmitriy Buyanov, Alexander Yu. Gerasimenko, Dmitry Repin

**Affiliations:** 1 Department of Informatics, Bioengineering, Robotics and System Engineering (DIBRIS)б, University of Genoa, 16146 Genoa, Italy; lisa.reganova@gmail.com; 2 Functional Neurophysiology Laboratory, Autonomous Noncommercial Organization National Technology Initiative University 2035”, 121205 Moscow, Russia; ks.p.solo@gmail.com; 3 Tri-Institutional Center for Translational Research in Neuroimaging and Data Science (TReNDS), Georgia Institute of Technology, The Georgia State University, Atlanta, GA 30303, USA; 4 Institute of Biomedical Systems, National Research University of Electronic Technology (MIET), Zelenograd, 124498 Moscow, Russia; buyancik@gmail.com; 5 Medical Computer Systems Ltd., Zelenograd, 124460 Moscow, Russia; 6 Institute for Bionic Technologies and Engineering, I.M. Sechenov First Moscow State Medical University, 119991 Moscow, Russia; 7 Improbability Foundation, Rue De-Candolle 19, CH-1205 Geneva, Switzerland

**Keywords:** EEG, fNIRS, functional connectivity, EMS, hypoxia, cognitive functions

## Abstract

Objectives: This study describes the effects of interval hypoxic training and electrical muscle stimulation (EMS) technology on human productivity with the following metrics: biochemical indices, cognitive abilities, changes in oxygenated (HbO) and deoxygenated (Hb) hemoglobin concentrations over the prefrontal cortex, and functional connectivity via electroencephalography (EEG). Methods: All measurements according to the described technology were made before the start of training and one month later, right after it ended. The study involved middle-aged Indo-European men. Specifically, there were 14, 15, and 18 participants in the control, hypoxic, and EMS groups, respectively. Results: EMS training improved reactions and nonverbal memory but decreased attention scores. Functional connectivity decreased in the EMS group while it increased in the hypoxic group. A result of the interval normobaric hypoxic training (IHT) was significantly improved contextual memory, with a *p*-value = 0.08. Conclusions: It was found that EMS training is more likely to cause stress on the body than positively affect cognitive functions. At the same time, interval hypoxic training can be considered a promising direction for increasing human productivity. The data obtained during the study can also help in the timely diagnosis of insufficient or overestimated indicators of biochemistry.

## 1. Introduction

In today’s world, it is necessary to process large volumes of information on a daily basis. This causes continuous stress and reduces overall energy and productivity levels [1]. Exercise is known to have numerous neuroprotective effects and the ability to improve higher cognitive functions, especially with regard to memory and learning processes [2]. For centuries, researchers have tried to elucidate the mechanisms of the relationship between physical health and cognitive abilities. Numerous studies have shown that physical activity has a number of sustained effects on the brain, such as improved memory [3], mood [4], plasticity [5], and learning abilities [6]. In addition, exercise has antidepressant effects [7] and counteracts disease or age-related mental impairment, including neurodegenerative diseases such as Alzheimer’s disease [8].

There are studies supporting improvements in episodic memory [9] and an increase in hippocampal size with aerobic exercise [10]. For example, dancing is an effective way to improve cognitive function, inducing brain plasticity at both structural and functional levels [11]. At the same time, resistance training has been proven to be more effective in improving aspects of cognition than aerobic exercise [12].

EMS is a method of muscle contraction involving the use of electric impulses. The impulses are produced by the generator of the myostimulator and are transmitted through electrodes to the surface of the skin near the muscles to be stimulated. The electrodes are usually glued to the skin or placed in special compartments in clothes. The impulses simulate the action potential of the central nervous system, causing the muscles contraction. Electrical muscle stimulation (EMS) is mainly used for muscle rehabilitation in partially or for fully immobilized people, for strength training and recovery in athletes and healthy people, and for assessments of muscle tissue condition [13].

A decrease in oxygen levels in individual tissues is known as hypoxia. Although it most commonly has a negative effect on health, interval normobaric hypoxic training (IHT), on the contrary, is used to increase the body’s endurance [14]. Usually there are two types of such training methods: (1) the participant is at rest, alternating putting on and taking off the hypoxic mask; (2) IHT during exercise. The first one is most often performed in order to stimulate altitude acclimatization, and the second one to intensify the training load by training at low altitudes in order to provide high-quality training. Levine claims that the first method shows the best results in athletes [15]. Athletes show an improvement in performance, the mechanism of which is primarily an increase in erythropoietin, leading to increases in red blood cell mass, maximum oxygen consumption (VO_2max_), and running performance.

Despite the long-standing conception that the number of neurons in the brain does not change after intrauterine and neonatal development, it has been shown [16] that new neurons can also be formed in adulthood. This happens during a process known as neurogenesis, which can mitigate the deleterious effects of neurodegeneration. The family of proteins that maintain the viability of neurons are also responsible for their development, which are called neurotrophins. One of the most important representatives, brain-derived neurotrophic factor (BDNF), helps maintain the survival of existing neurons and stimulates the growth and differentiation of new ones through axon and dendrite outgrowth. In addition, previous studies revealed the important role of this protein in synaptic plasticity and memory consolidation [17].

Skeletal muscles are known to be some of the sites of BDNF synthesis and accumulation [18]. When muscles contract, BDNF is released into the blood. Then, moving along the blood vessels, the growth factor crosses the blood–brain barrier and reaches the brain. Previous studies have shown a short-term increase in the level of BDNF in elderly patients undergoing EMS therapy [19]. In addition, another group of scientists [20] have shown that the combination of neuromuscular electrical stimulation (NMES) and voluntary contractions leads to changes in functional connectivity and an increase in plasticity.

Interval hypoxia can cause a serotonin-dependent increase in BDNF synthesis in the ventral spinal segments containing the diaphragmatic nucleus [21]. It was also determined that the magnitude of this increase in BDNF correlated with the phrenic long-term facilitation (pLTF) value. Moreover, decreases in attention, memory, and executive functions were found during hypoxic training [22]. The type of cognitive change is strongly dependent on the type of cognitive task, type of exercise, intensity of exercise, type of training, and level of hypoxia.

However, available studies have separately assessed physiological and cognitive markers and have been conducted on the diseased and athletes (in the case of EMS), or in the case of IHT were very dependent on training parameters [13,15,19,22]. Therefore, our first goal was to comprehensively study the effect of EMS and IHT training offered to ordinary healthy people in specialized fitness centers or clinics. In addition, the second aim was to find correlations between cognitive parameters (attention, reaction rate, etc.), biochemical indices, and the functional connectivity of the brain.

## 2. Materials and Methods

### 2.1. Participants

Forty seven Indo-European males, aged 25–47, were recruited as participants in the study. The participants’ physical activity level was defined as the average number of training sessions per week (strength or cardio) longer than 30 min. All participants performed an office type of work, which did not affect their level of physical activity during the day. The inclusion criteria were: (1) male gender; (2) age 25 to 50 years; (3) the ability to attend training regularly. Reasons for not participating were: (1) chronic diseases; (2) too high or low levels of body mass index (BMI), 18 < BMI < 27; (3) regular EMS or hypoxic training sessions. Participants were randomly assigned to groups, taking into account the uniformity of the physical parameters described in Table 1.

One participant from the hypoxic group was engaged in mountain climbing, while the others had no experience of being under hypoxia. The participants were asked not to change the type and level of sport activity during the experiment.

The control group did not participate in specific training. However, all participants in the control group, as well as all other participants on a daily basis, twice a day (morning and evening), underwent a five-minute heart rate variability (HRV) scan using the Welltory application and noted tags to assess their subjective state.

The study was conducted in October and lasted a month; during this period the daylight hours decreased by more than two hours and the average temperature dropped from 10 °C to 2 °C. The data were collected at the functional neurophysiology laboratory of the autonomous noncommercial organization “National Technology Initiative University 2035” (Moscow, Russia). The interdisciplinary data collection and processing team included biophysicists, biomedical engineers, and medical professionals with advanced degrees.

### 2.2. Ethics

The research was conducted ethically in accordance with the World Medical Association Declaration of Helsinki. The research was approved by the local ethics committees of I.M. Sechenov First Moscow State Medical University (protocol No_23-18 on 09.05.2022). Informed consent was obtained from all participants.

### 2.3. Trainings

#### 2.3.1. Hypoxic

Interval hypoxic training was conducted using the Hypoxico Everest Summit II Altitude Generator (Hypoxico Inc., New York, NY, USA). The composition of the inhaled mixture corresponded to an altitude of 6000 m. Training sessions were held three times a week. The selection of the program was carried out based on an initial test to restore saturation after hypoxia. The minimum training duration was 40 min and the maximum was 1 h. Each participant had 12 training sessions. During the training sessions, the participants were seated in a relaxed posture, allowing them to remain at rest. The duration of each block of training varied from 30 s to 5 min, depending on the participant’s initial training level, and increased incrementally with each subsequent training session.

#### 2.3.2. EMS

XBody Actiwave (XBODY International Kft., Budapest, Hungary) equipment was used for the EMS training. The training program was developed by an XBODY International Master Trainer in EMS technology, and included 8 training sessions. The intervals between trainings were not less than three days and not more than five days. The sessions differed in duration but did not exceed 40 min. Each training session ended with a lymphatic drainage massage.

The training program took into account the average general physical condition of the participants and the smooth adaptation to the exercise. In the protocol, the first workouts were aimed at smoothly bringing the participants into contact with the electrical impulse and activating the cortex and stabilizer muscles. The first training session was of low intensity, consisting of a “strength” and “lymphatic drainage” program, basic exercises without complex movements, and activating more superficial muscles (oblique abdominal muscles, lumbar muscles, straight thigh muscles, small and middle gluteal muscles, muscles of the posterior surface of the thigh). On a 10-point scale of pulse intensity sensitivity, this was in the range of 5–6. The second workout was of medium intensity, and on the 10-point scale of sensitivity of the intensity of the impulse it was in the range of level of 6–7. Basic exercises with elements of more complex movements activate the deeper muscles of the stabilizers (transversal abdominal muscles; multiseptal muscles of the spine; iliopsoas-lumbar muscles). The third workout was above average intensity, consisting of three programs: “strength”, “cardio”, and “lymphatic drainage”. On a 10-point pulse intensity sensitivity scale it was at level 8. The exercises were multi-jointed with rotation, which ensured the inclusion of the deep stabilizer muscles, as well as the stabilizer muscles of the shoulder joint. The “cardio” program allowed an increase in the intensity of the circulation. The fourth and fifth workouts were of intensified intensity, due to changes of the pulse time, pulse pauses, and pulse frequency (red, intermediate, and white fibers participate in the process of optimal tetanus). The sixth and seventh training programs were even more intensive, due to changes in the impulse time and pauses, impulse frequency, and amplitude of the modulated impulse. The eighth and final training session was of high intensity due to changes in the impulse time, impulse pauses, and impulse frequency. The training was aimed at strengthening the muscles and blood and lymphatic flow.

### 2.4. Cognitive Tests

#### 2.4.1. Cognifit

The participants’ cognitive abilities were assessed using the cognitive assessment battery for the coordination (CAB-CO) test on the Cognifit platform (CogniFit Ltd., Nazareth Illit, Israel). A total of 28 parameters were measured with it. The participants could take it in the laboratory or at home (the same for measurements at the beginning and at the end of the experiment).

#### 2.4.2. 2-Back

During the recording of functional near-infrared spectroscopy (fNIRS) values, the participants took a 2-back test created for the experiment on the PsyToolKit platform [23]. It consisted of one training session and five control blocks. Twenty-five stimuli were presented in each control block. In the training and test block, the stimulus lasted 1 s, and the pause between stimuli was 2 s. The pause between blocks was 17 s. The total duration of the control part was 5 min and 20 s.

### 2.5. HRV and Biochemical Parameters

The participants in all groups took HRV measurements twice a day during the experiment using the Welltory app (Welltory Inc., Redwood City, CA, USA) and a smartphone camera or Apple Watch (Apple Inc., Cupertino, CA, USA). The tags noted the presence of exercise that day. Before and after the experiment, the participants had blood tests for hormones (adrenaline, norepinephrine, serotonin, and dopamine), a lithium test, a biochemical analysis (22 indices), and a BDNF test.

### 2.6. Brain Activity

#### 2.6.1. EEG Data Recording and Processing

Four electroencephalographic (EEG) recordings, each lasting 3 min, were made for each participant—two before the experiment (first recording with eyes opened, then with eyes closed) and two after. All EEG signals were recorded with a NeuroPlay-8Cap (Neurobotics Ltd., Moscow, Russia; self-noise 3–4 mV) [24], which consisted of 8 surface electrodes (F3, F4, C3, C4, P3, P4, O1, O2) mounted on a cap following the International 10–20 positioning system. The ground and reference electrodes were A2 electrodes. The sampling frequency was 125 Hz. The data were preprocessed using the SciPy library in Python [25]. The signal was divided into periods of 1 s duration; periods with a signal amplitude above 100 µV were excluded. The spectral bands used for further signal processing were delta (0.5–4 Hz), theta (4–8 Hz), alpha (8–12 Hz), beta-1 (12–18 Hz), beta-2 (18–30 Hz), and gamma (30–48 Hz).

The phase lag index (*PLI*) and coherence (*COH*) methods were used to study the connectivity.

The coherence was considered as:

(1) $${COH}_{ij}^{2}\left(w\right)=\frac{E[C_{ij}^{2}(w)]}{E[C_{ii}^{2}(w)]*E[C_{jj}^{2}(w)]},$$

where *E*[] is the epoch average, $C_{ij}(w)$ is the Fourier transform of the cross-correlation between EEG nodes (node *i* and node *j*), and $C_{ii}(w)$ is the co-spectrum. The coherence was calculated in 10 s overlapping windows in the frequency domain. Then, the coherences of all epochs were averaged over time.

The phase lag index was determined as:

(2) $$PLI=\left|<sign[∆\varphi (t_{k})]>\right|,$$

where ∆*φ*(*t_k_*), *k* = 1… N is the time series of signal phase differences. The *PLI* ranges from 0 to 1: 0 ≤ *PLI* ≤ 1. A *PLI* equal to zero indicates either no coupling or coupling with a phase difference centered around multiples of *π* angles. A *PLI* equal to 1 indicates perfect phase synchronization at a value of ∆, other than 0 and multiples of *π*. The stronger this nonzero phase synchronization, the greater the *PLI*.

The frontal characteristics were calculated using electrodes F3, F4, C3, and C4. The interhemispheric connectivity was considered as the average connectivity between the pairs of electrodes C3-C4, P3-P4, C3-P4, and C4-P3. The sagittal connectivity was considered as the average connectivity between the pairs of electrodes F3-O1, F4-O2, F3-O2, and F4-O1.

#### 2.6.2. fNIRS Data Acquisition and Processing

The fNIRS measurements were conducted with an NIRS4 brain and body spectrometer (Medical Computer Systems Ltd., Zelenograd, Moscow, Russia, resolution 24 bits) [26] using two 4-channel arrays of optodes (1 light source and 4 detectors in each device), covering the frontal and prefrontal areas.

Each device was square-shaped with a diagonal length of 5 cm. At the vertices of the square there were detectors, and in the center there was a source. The device was installed in such a way that the diagonal of the first square fell on positions F6 and Fp2, and the diagonal of the second square fell on positions F5 and Fp1. Near-infrared light was used at two wavelengths (770 and 850 nm). Changes in the concentrations of HbO and Hb were continuously recorded throughout the 2-back test using NIRS4LSL software (Medical Computer Systems Ltd., Zelenograd, Moscow, Russia). Signals from 8 NIRS channels were acquired at a sampling rate of 10 Hz.

The interaction of the radiation with the medium was described using the Beer–Lambert law. Data from the spectrometers were sent to the NIRS4LSL Software-17154 (Medical Computer Systems Ltd., Zelenograd, Moscow, Russia). Next, using the lab streaming layer protocol [27], the data were sent to the open source software LabRecorder [28]. The data received from LabRecorder in .xdf format were processed in Matlab R2020a (The Mathworks, Inc., Natick, MA, USA). Oxygenated and deoxygenated hemoglobin values were converted to total hemoglobin (HbT) and tissue saturation index (TSI) values.

Hb + HbO = HbT,(3)

(HbO/HbT) × 100 = TSI.(4)

For each stage of resting and testing, the arithmetic mean of the oxygenated and deoxygenated hemoglobin and the tissue saturation index were calculated. During data processing, artifacts associated with muscle activity in the frontotemporal region were found. These noisy data were excluded from the considered sample. Despite the obvious advantages of fNIRS, it was necessary to pay attention to possible limitations during the examination. A significant error in the measurement result can be introduced by tissues located between the sensor and the object of study, by the air gap, and by the mobility of the sensor.

### 2.7. Statistical Analysis

With the exception of the fNIRS data, all data processing and visualization were performed using Python (Version 3.8.8). To analyze the biochemistry and cognitive parameters, appropriate libraries were used such as Pandas (Version 1.0.1), NumPy (Version 1.18.1), SciPy (Version 1.4.1), Plotly (Version 4.14.3), and MatplotLib (Version 3.1.3). The delta of the changes in all parameters was measured separately for each participant (by subtracting the value after from the value before). A further analysis was performed by comparing the distribution of these values in the three groups. An evaluation of the distribution normality (for the values obtained before the experiment) was performed both visually (by plotting histograms and kernel density estimates) and using Shapiro–Wilk’s statistical test (scipy.stats.shapiro). Not all metrics passed the normality test; therefore, the nonparametric Mann–Whitney test was used for pairwise comparisons. Holm’s correction (statsmodels.stats, multitest.multipletests) was applied for multiple hypothesis testing. A Kolmogorov–Smirnov test with Bonferroni correction and a Tukey test were used to check whether all three treatments came from the same distribution. It was found that there were significant differences in the RBC, hemoglobin, and hematocrit levels between the EMS and hypoxic groups before the beginning of the experiment. Therefore, these parameters were not used for the next comparison. Furthermore, the statistical tests did not reject the null hypothesis for the cognitive parameters. The power analysis was performed with the G*Power 3.1 software. Only results with a large effect size (>0.75) were included in the article.

The Spearman and Kendall coefficients (stats.spearmanr, stats.kendalltau) were calculated to find correlations under the assumption of nonlinear dependence. The significance level was chosen to be 0.05 for all tests. For the processing and postprocessing of EEG data, in addition to the above, the library mne (version 0.22.0) was used. The calculated coherence coefficients and phase lag index were subjected to the same test for normality and a multiple analysis of variance (ANOVA).

The processing of the participants’ cardiac activity data collected using the Welltory 3.0 or Apple WatchOS 8 was performed using the HrvAnalysis (Version 1.0.4) and PyHrv (Version 0.4.0) libraries. As a result of the signal cleaning and processing, we obtained temporal, geometric, frequency, and nonlinear metrics. Using the Sklearn library, regression models of changes in these metrics and their confidence intervals were constructed for the groups. Next, the regression coefficients were used for pairwise comparisons.

## 3. Results

**Figure 1:**
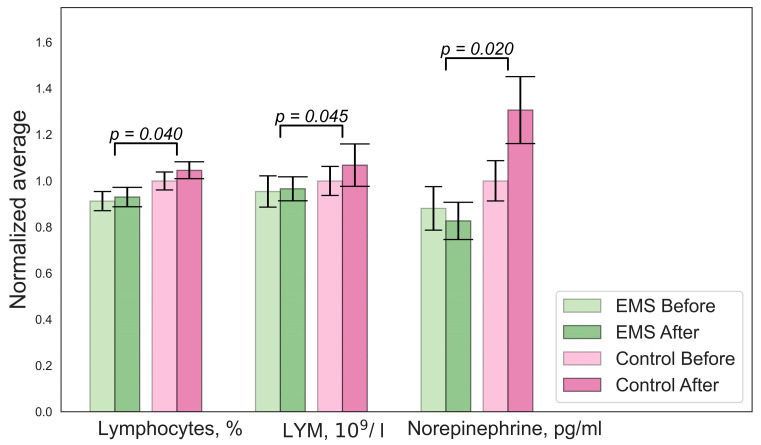
Significant changes in biochemistry parameters for the EMS group. Error bars indicate the standard error of the mean (SEM).

**Figure 2:**
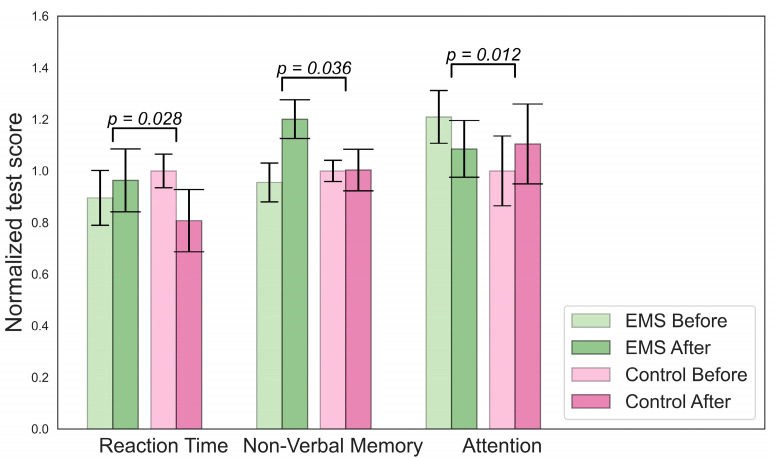
Significant changes in cognitive parameters for the EMS group.

**Figure 3:**
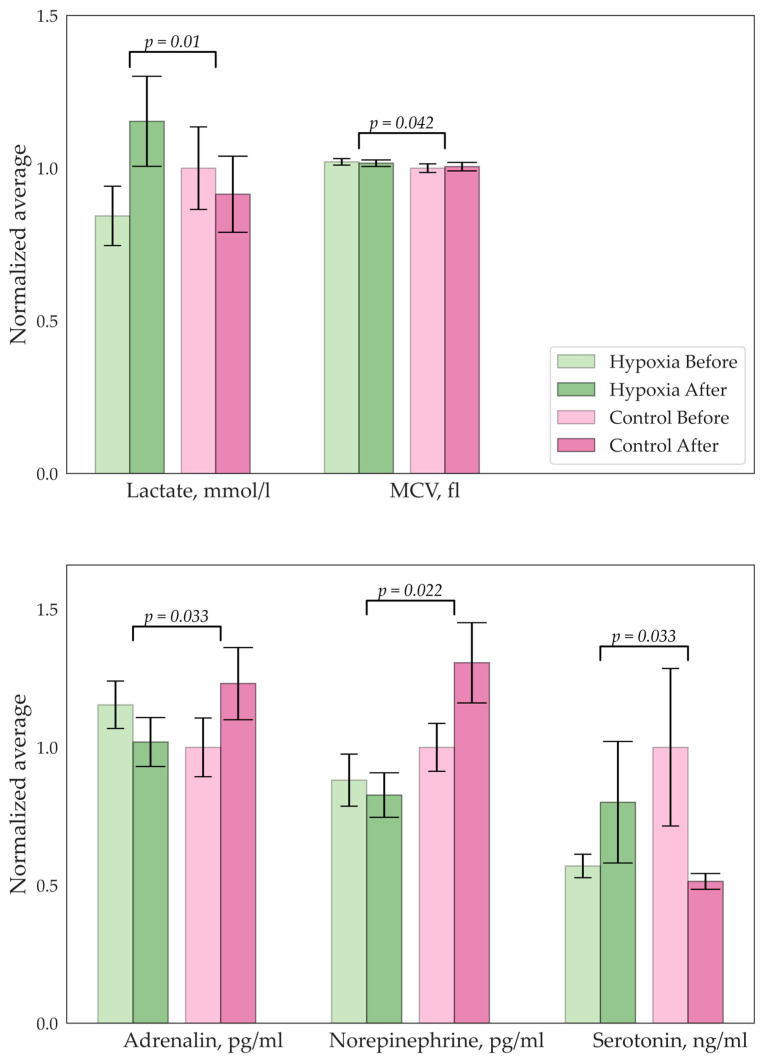
Significant changes in biochemistry parameters for the hypoxic group.

**Figure 4:**
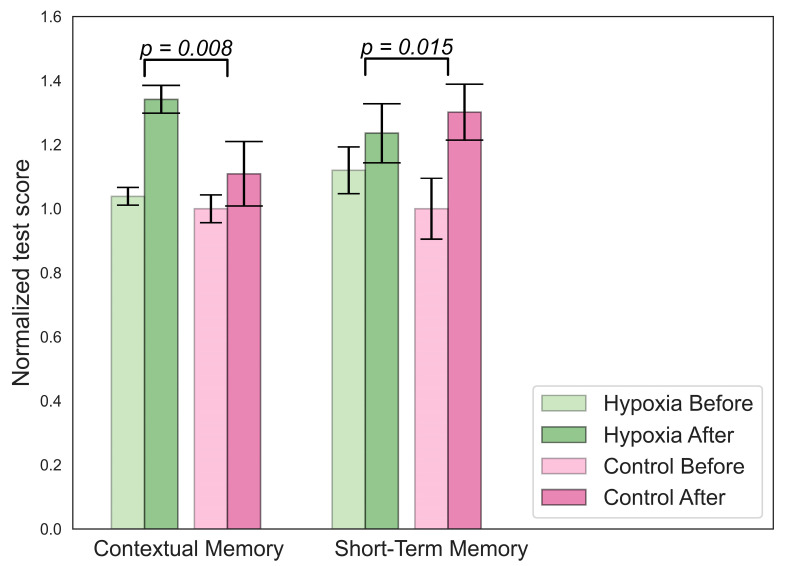
Significant changes in cognitive parameters for the hypoxic group.

Figure 1, Figure 2, Figure 3 and Figure 4 illustrate the results of the statistical analysis. The graphs below show the normalized mean values of the indicators for the group before and after. The error bars indicate the standard error of the mean (SEM). Figure 1 shows changes in the biochemistry of the EMS group.

Figure 2 describes changes in cognition for the EMS group. To determine which changes were significant, we compared the delta for the EMS and hypoxia group (value after minus value before) with the change in the control group.

There were significant differences in the concentrations of lactate, mean cell volumes (MCVs), and numbers of hormones in the hypoxic group, as shown in Figure 3. A decrease in lymphocyte and norepinephrine concentrations was observed in the EMS group. No difference in BDNF levels was found. An analysis of the Cognifit test scores revealed a significant main effect of the group on the cognitive parameters. The EMS-trained participants had better reactions and nonverbal memory but they scored lower on the attention tests.

An increase in contextual memory was found in the hypoxic group, as shown in Figure 4. The participants from this group also showed higher scores on the short-term memory test compared to the beginning, but this increase was smaller than in the control group.

### 3.1. Welltory Data

The data in Table 2 refer to the changes in the slope coefficient for the linear regression model approximating the changes in participants’ metrics for the morning measurements during the experiment. The change in slope was considered in comparison with the change in slope in the control group. The table presents only significant results.

The analysis showed a shift in the balance of high-frequency power (Hf) and low-frequency power (Lf) intervals towards LF in the EMS group. In the hypoxic group, the participants showed a decrease in the nearest neighbor index (NNi) and increase in the min HR trends.

### 3.2. EEG Functional Connectivity

**Figure 5:**
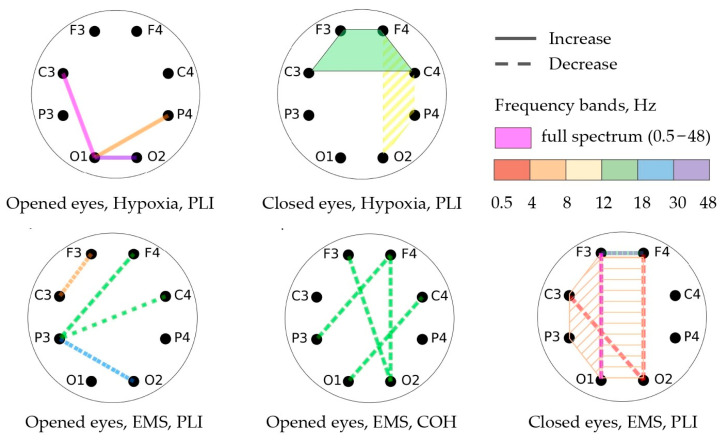
Significant changes in FC between different channels.

Table 3 and Table 4 and Figure 5 showcase the findings of the functional connectivity (FC) changes during open-eye and closed-eye states, as measured using the phase lag index (*PLI*) and coherence (*COH*) methods. The changes in FC are depicted by lines, with dotted lines indicating a decrease and solid lines indicating an increase. The frequency ranges in which these changes occur are shown through different colors. If the connectivity occurs across more than two channels, a polygon is used to indicate this.

The participants in the EMS group experienced a decline in functional connectivity during the open-eye state, while the participants in the hypoxic group showed an increase in functional connectivity. In particular, the EMS group showed a decrease in coherence in the beta-1 frequency range for several channel pairs with open eyes and a decrease in *PLI* for the central and left regions in the theta range. On the other hand, the hypoxic group demonstrated an increase in *PLI* in the frontal region with closed eyes in the beta-1 frequency range.

In addition to the results of the post hoc power analysis, we would also like to highlight that the sample size used in this study was sufficient to detect a statistically significant effect size. The analysis conducted using the “G*Power 3.1” software showed that the current sample size had an adequate power level (1–β > 0.8) at an alpha level of 0.05. This indicates that the results of this study are reliable and can be used to draw meaningful conclusions.

### 3.3. fNIRS

**Figure 6:**
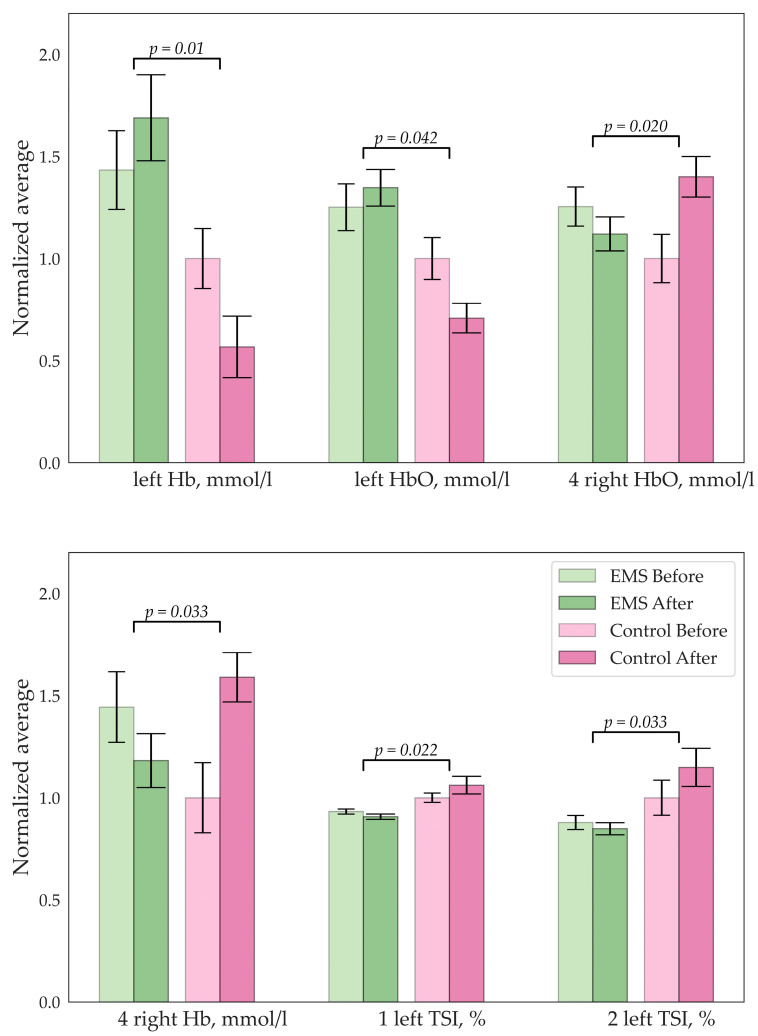
Significant changes in concentration of the Hb/HbO (mmol/L) and tissue saturation index (%); digits indicate the number of the sensor.

Significant changes were detected only in the group with EMS training. Increase in the hemoglobin and oxyhemoglobin concentrations were observed, as well as a decrease in TSI in the left prefrontal cortex. On the contrary, a decrease in hemoglobin concentration was observed in the right prefrontal cortex. These results are represented in Figure 6.

### 3.4. Correlations

**Figure 7:**
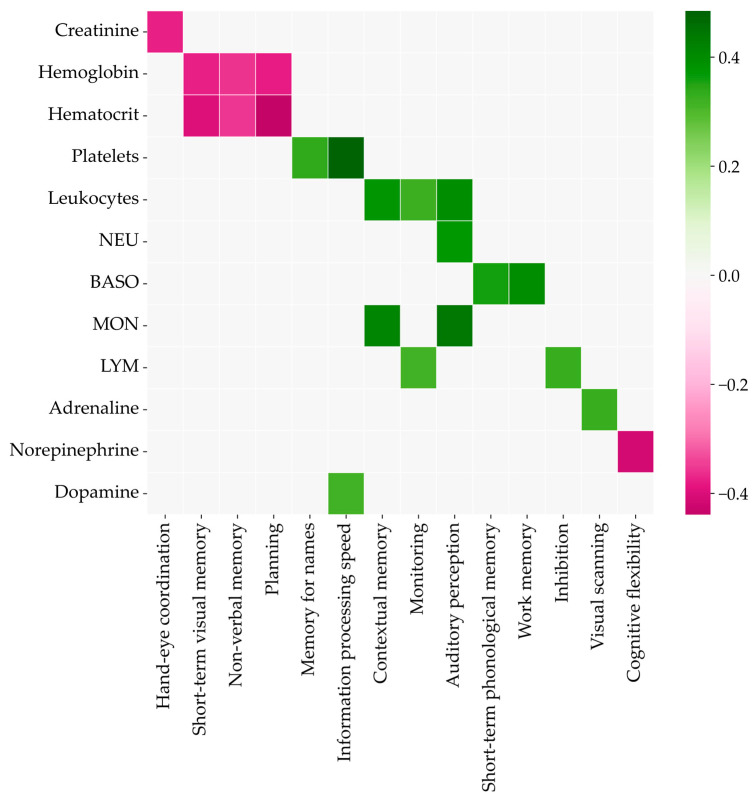
Spearman’s rank correlation coefficients between cognitive and biochemical parameters.

The correlations of biochemistry and cognitive parameters were calculated for pretraining data to exclude their influence. The Spearman coefficients are shown in color on the heatmap in Figure 7. The higher concentrations of creatinine, hemoglobin, hematocrit, and norepinephrine meant lower scores for the corresponding cognitive abilities. Other biochemistry parameters were positively correlated with the relevant test scores.

**Figure 8:**
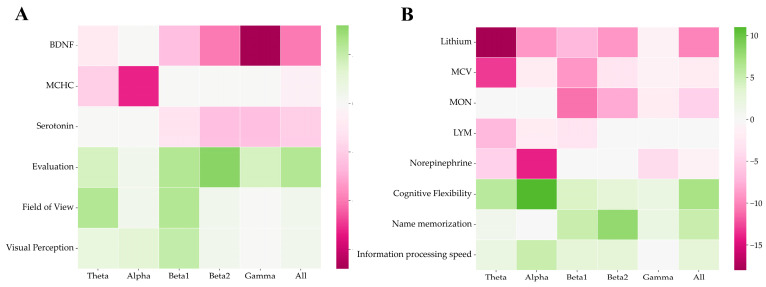
Spearman’s rank correlations between cognitive and biochemical parameters and FC in the open-eye state (**A**) and in closed-eye state (**B**) before the experiment. The FC was calculated using the coherence method.

We also searched for possible correlations of the biochemistry indices, cognitive tests, and functional connectivity of the EEG results (according to the data before training). The results are shown in Figure 8, where pink means a negative correlation and green means a positive correlation. The color shade indicates the number of channel pairs for which the FC is correlated with the parameter in this band (detailed Spearman’s rank correlations are represented in the Supplementary Materials). It was found that in the open-eye state, BDNF was negatively correlated with connectivity at high frequencies (beta and above), with particularly strong correlations with FC in the gamma band. Estimation and visual perception were positively correlated with connectivity in many channels and in all frequency bands. Overall, there was a trend in the open-eye condition toward a positive correlation of connectivity and cognitive test scores and a negative correlation of connectivity with biochemistry scores. A causal analysis was performed for all measures of biochemistry and cognitive tests. The PC and GES algorithms from the python causal-learn package were used for the analysis (causal graph equivalence classes are represented in the Supplementary Materials).

As for the closed-eye state, the trend toward a negative correlation of functional connectivity with biochemistry indices and a positive one with cognitive tests was observed here as well. Thus, lithium was negatively correlated in the alpha, beta, and especially theta bands, the mean erythrocyte size (MCV) in the theta and beta bands, and the monocyte concentration only in the beta band. The norepinephrine levels showed a negative correlation with connectivity in the alpha band. In contrast, a positive correlation was found for cognitive flexibility and FC at low frequencies in the alpha and theta bands, and the best ability to remember names correlated with coherence in the beta range.

## 4. Discussion

The primary purpose of this study was to comprehensively study the effects of EMS and IHT training offered to ordinary healthy people in specialized fitness centers or clinics. The secondary aim was to find correlations between cognitive parameters (attention, reaction rate, etc.), biochemical indices, and the functional connectivity of the brain. The main findings were as follows: (1) It was determined that EMS training puts the body in a state of stress rather than improves cognitive abilities. (2) Interval hypoxic training has a positive effect on cognitive abilities and can be considered a promising direction for increasing human productivity. Indirect indicators for measuring productivity were used, including a set of biochemical indicators, HRV indicators (stress and recovery levels), cognitive characteristics (attention, memory, thinking), and brain activity (oxygen consumption efficiency and functional connectivity). (3) Poor short-term and working memory correlates with low levels of basophilia. (4) Correlations of functional connectivity with biochemical and cognitive tests were obtained.

Based on the results for changes in biochemical parameters and HRV, we may conclude for the group with EMS training that the balance of the autonomic nervous system is shifted towards the sympathetic nervous system. A decrease in HFnu and an increase in LFnu were revealed in the participants of this group. According to Shaffer and Ginsberg’s study [29], the power of LF waves reflects the sympathetic nervous system function and the HF waves reflect the parasympathetic nervous system. Additionally, the LFnu index is considered an indicator of physiological stress. A high LF/HF ratio indicates sympathetic dominance, which occurs when we engage in a fight-or-flight response or parasympathetic detachment. Another indicator of physiological stress in the body is a decrease in lymphocyte levels. These are some of the main cells of the immune system responsible for antibody production and cellular immunity. Thus, the observed decrease in lymphocyte levels leads to decreases in immunity and antibody production. Previous research studies have found that stress leads to an increase in the activity of the hypothalamic–pituitary–adrenal (HPA) axis, which leads to increased release of glucocorticoids from the adrenal cortex [30]. Moreover, it was shown that the administration of hydrocortisone at the time of its maximum concentration in the plasma (in the afternoon) leads to a decrease in reaction time, which was also confirmed in this work [31].

An increase in the lactate level is the expected adaptive reaction of the organism for the group with hypoxic training. The IGT-mediated activation of glycolysis and suppression of the citric acid cycle (CAC) are critical adaptive responses in the early stage of hypoxia [32,33]. In other words, an anaerobic glucose oxidation pathway is triggered, resulting in pyruvic acid reduction to lactic acid (lactate). The mechanism is similar to lactate production during stress testing. At a low load, the concentration of lactate in the blood (an indicator characterizing the activity of glycolysis) does not change with increasing power, but at a certain load value it begins to grow. As a criterion for the activation of glycolysis, a fixed value of the blood lactate concentration of 2 mmol/L is often used as the upper limit of the norm for the concentration of lactate at rest [34], while 4 mmol/L is the threshold level, which corresponds to the population average value of the maximum concentration of lactate, at which there is a balance between the release of glycolysis products into the blood and their utilization [35].

Another protective mechanism is an increase in heart pumping cycles. Participants with IHT also had higher mean and minimum HRs. In addition, the changes in the hormones adrenaline and noradrenaline confirmed the results of previous investigations showing that norepinephrine levels in the urine and serum increase significantly during high-altitude stays, while adrenaline seems to be less affected by this effect [36]. Improved memory was confirmed in a study in mice [37]. This paper demonstrates the expansion of past results to humans as well.

The negative correlation of hemoglobin and hematocrit with the ability to remember extends the results of the previous research showing that a higher hemoglobin level is associated with memory impairment in the long term [38]. Rihet et al. [39] gave a vague suggestion that the dopaminergic system plays a role in sensory processing, possibly acting at the level of arousal. This study confirmed this assumption and points to a positive correlation.

It has been previously shown that beta rhythm activity signifies being in a state of concentration [40]. Therefore, the positive correlation of FC in the beta range with remembering names suggests support for the idea that this process does require a person to concentrate on a physiological level. During meditation, in-person activity of the theta and alpha rhythms is observed. From the correlation of coherence in these bands with cognitive flexibility, it can be suggested that meditation exercises can help improve cognitive flexibility. The negative correlation of serotonin with coherence at high frequencies may imply a decreased ability to concentrate and solve complex tasks when serotonin levels in the blood are high.

It is noteworthy that significant variations were detected in biochemistry and cognitive measures among the control group. This may be attributed to seasonal fluctuations, as the study took place during autumn and spanned a one-month duration. During this period, the day length was reduced by over two hours and the average temperature dropped by 8 °C. There is a growing body of evidence suggesting that seasonality can impact both biochemistry and cognitive measures [41,42]. Specifically, other studies have reported decreased serotonin levels among control groups during the autumn to winter transition, as observed in our study [43,44].

### Limitations and Future Research

One of the limitations of our study was the lack of a sham procedure, which may limit the interpretation of the results and raise concerns about the validity of the findings. A sham procedure would provide a more controlled environment to differentiate between true treatment effects and placebo effects. Although we did have a control group in our study, it is important to acknowledge the limitations of not having a sham procedure.

Another limitation of our study was the narrow inclusion criteria, which only allowed for Indo-European men, of average age and body mass index, with no significant athletic experience to participate. This restricted participant pool may not be representative of the general population, meaning the results may not be generalizable to other populations. Additionally, a small sample size may lead to bias and affect the reliability of the results. This limitation needs to be considered when interpreting and applying the results of our study. Therefore, future studies should explore the use of a sham procedure and extend the inclusion criteria to increase the study’s universality.

## 5. Conclusions

In this paper, we conducted a study testing the hypothesis about the effect of EMS and interval hypoxic training on human productivity biomarkers. The results of changes in biochemical parameters and the analysis of NN intervals indicated a shift in the autonomic balance toward the sympathetic nervous system in the group with EMS training. At the same time, a decrease in functional connectivity of EEG across many channels was observed.

Decreases in attention and improved reaction times were detected. The group with IHT had natural adaptive changes in biochemistry and HRV to the conditions of oxygen deficiency. In addition, statistically significant increases in the functional connectivity of several channels were detected in this group. Additionally, these participants showed an improvement in contextual memory but a decrease in short-term memory. Moreover, correlations were found between biochemistry and cognitive test scores. These results may help in the timely diagnosis of insufficient or overestimated biochemistry scores. For example, poor short-term and working memory correlate with low basophilic levels.

In addition, correlations of functional connectivity with biochemistry and cognitive tests were obtained. From these data, it is possible to infer ways in which certain measures can be improved. For instance, improvements in contextual flexibility can be achieved by practicing meditation. In general, training with EMS is more likely to put the body into a state of stress, and interval hypoxic training can be considered a perspective direction for increased productivity.

## Figures and Tables

**Table 1:** The subjects’ characteristics.

Characteristic	EMS (N = 18)	Hypoxia (N = 15)	Control (N = 14)
Age, years	33.3 ± 3.6	33.9 ± 3.9	35.2 ± 3.8
Height, cm	182 ± 6	178 ± 5	181 ± 8
Weight, kg	79 ± 12	76 ± 11	78 ± 10
BMI, kg/m^2^	23.7 ± 3.5	24.0 ± 1.7	24.4 ± 3.1
Physical activity (out of 5)	2.4 ± 2.3	2.6 ± 1.6	3.1 ± 1.7

**Table 2:** Significant changes in RR interval features.

Parameter	Group	Type of Change	*p*-Value	Effect Size
Total Power	Hypoxia	Decrease	0.046	0.82
VLf	Hypoxia	Decrease	0.026	0.86
Range NNi	Hypoxia	Decrease	0.011	0.83
NNi 50	Hypoxia	Decrease	0.035	0.75
Min HR	Hypoxia	Increase	0.016	1.1
HfNu	EMS	Decrease	0.028	0.93
LfNu	EMS	Increase	0.028	0.92
Csi	EMS	Increase	0.010	1.1

VLf—very low frequency; HfNu—normalized high-frequency power; LfNu—normalized low-frequency power; Csi—cardiac sympathetic index.

**Table 3:** Significant changes in FC for the hypoxic group.

Channels	Bands, Hz	Condition	Method	*p*-Value	Effect Size	Power	Type of Change
P4-O1	4–8	OE	*PLI*	0.036	0.81	0.95	Increase
O2-O1	30–48	OE	*PLI*	0.011	0.90	0.98	Increase
C3-O1	0.5–48	OE	*PLI*	0.014	0.89	0.98	Increase
Right	8–12	CE	*PLI*	0.041	0.78	0.93	Decrease
Frontal	12–18	CE	*PLI*	0.008	0.96	0.99	Increase
O2-F4	30–48	CE	*COH*	0.032	0.85	0.97	Decrease

OE—the state with opened eyes; CE—the state with closed eyes; *PLI*—the phase lag index; *COH*—EEG coherence.

**Table 4:** Significant changes in FC for the EMS group.

Channels	Bands, Hz	Condition	Method	*p*-Value	Effect Size	Power	Type of Change
F3-C3	4–8	OE	*PLI*	0.031	0.83	0.96	Decrease
F4-P3	12–18	OE	*PLI*	0.042	0.76	0.92	Decrease
C4-P3	12–18	OE	*PLI*	0.044	0.79	0.94	Decrease
O2-P3	18–30	OE	*PLI*	0.016	0.97	0.99	Decrease
F4-P3	12–18	OE	*COH*	0.034	1.1	0.99	Decrease
C4-O1	12–18	OE	*COH*	0.049	0.97	0.99	Decrease
O2-F3	12–18	OE	*COH*	0.010	1.1	0.99	Decrease
O2-F4	12–18	OE	*COH*	0.010	1.1	0.99	Decrease
AVG	0.5–4	CE	*PLI*	0.001	1.25	0.99	Decrease
FO	4–8	CE	*PLI*	0.021	0.85	0.94	Decrease
Left	4–8	CE	*PLI*	0.019	0.86	0.97	Decrease
O2-C3	0.5–4	CE	*PLI*	0.016	0.96	0.99	Decrease
O2-F4	0.5–4	CE	*PLI*	0.010	0.96	0.99	Decrease
F4-F3	12–18	CE	*PLI*	0.041	0.74	0.89	Increase
F4-F3	30–48	CE	*PLI*	0.005	1.1	0.99	Decrease
F3-O1	0.5–48	CE	*PLI*	0.045	0.78	0.93	Decrease

## Data Availability

The data presented in this study are available on request from the corresponding author.

## Supplementary Materials

The following supporting information can be downloaded at: https://www.mdpi.com/article/10.3390/bioengineering10050536/s1.
