# Supplementary material for: Effects of Intermittent Hypoxia and Electrical Muscle Stimulation on Cognitive and Physiological Metrics
Source: Bioengineering (Basel). 2023 Apr 27;10(5):536. doi: 10.3390/bioengineering10050536 (PMC10215293; doi:10.3390/bioengineering10050536)
Supplement: Supplementary file 1 [file bioengineering-10-00536-s001.zip › bioengineering-2318366-supplementary-final-corrected.pdf]

bioengineering

Supplementary Materials

Effects of Intermittent Hypoxia and Electrical Muscle Stimulation on Cognitive and Physiological Metrics

Elizaveta Reganova [1,2], Ksenia Solovyeva [2,3], Dmitriy Buyanov [4,5], Alexander Yu. Gerasimenko [4,6,*] and Dmitry Repin [7]

1 University of Genoa, Department of Informatics, Bioengineering, Robotics and System Engineering (DIBRIS)б 16146 Genoa, Italy; lisa.reganova@gmail.com
2 Functional Neurophysiology Laboratory, Autonomous Noncommercial Organization National Technology Initiative University 2035", 121205 Moscow, Russia; ks.p.solo@gmail.com (K.S.); dr@improbability.foundation (D.R.)
3 Tri-Institutional Center for Translational Research in Neuroimaging and Data Science (TReNDS), Georgia Institute of Technology, The Georgia State University, Atlanta, GA 30303, USA
4 Institute of Biomedical Systems, National Research University of Electronic Technology (MIET), Zelenograd, 124498 Moscow, Russia; buyancik@gmail.com
5 Medical Computer Systems Ltd., Zelenograd, 124460 Moscow, Russia
6 Institute for Bionic Technologies and Engineering, I.M. Sechenov First Moscow State Medical University, 119991 Moscow, Russia
7 Improbability Foundation, Rue De-Candolle 19, CH-1205 Geneva, Switzerland
* Correspondence: gerasimenko@bms.zone

Table S1. Changes in the values of biochemical and cognitive parameters. The group in which there was a significant change compared to the control group is highlighted in bold.

Parameter	Hypoxia	Control	EMS
Lactate,mmol/l	**0.42 ± 0.17**	−0.12 ± 0.23	−0.04 ± 0.13
MCV, fl	**−0.40 ± 0.29**	0.45 ± 0.31	0.30 ± 0.21
Adrenalin, pg/ml	**−3.53 ± 3.51**	6.04 ± 3.46	2.27 ± 2.88
Serotonin, ng/ml	**10.73 ± 10.47**	−22.57 ± 13.38	−13.0 ± 9.46
Norepinephrine, pg/ml	**−15.25 ± 27.45**	86.21 ± 37.83	**−21.02 ± 30.41**
Lymphocytes, %	0.72 ± 1.63	1.88 ± 0.89	**−0.67 ± 0.91**
LYM, 10^9/l	0.03 ± 0.10	0.15 ± 0.14	**−0.12 ± 0.10**
Contextual Memory	**140.2 ± 19.83**	50.5 ± 28.97	93.13 ± 15.48
Short Term Memory	**57.0 ± 28.43**	148.9 ± 28.6	160.73 ± 53.54
Reaction Time	8.87 ± 38.73	−73.3 ± 27.25	**25.93 ± 53.0**
Non verbal Memory	25.2 ± 27.83	1.4 ± 20.58	**102.4 ± 45.14**
Attention	214.14 ± 108.36	55.62 ± 49.76	**−66.0 ± 65.83**

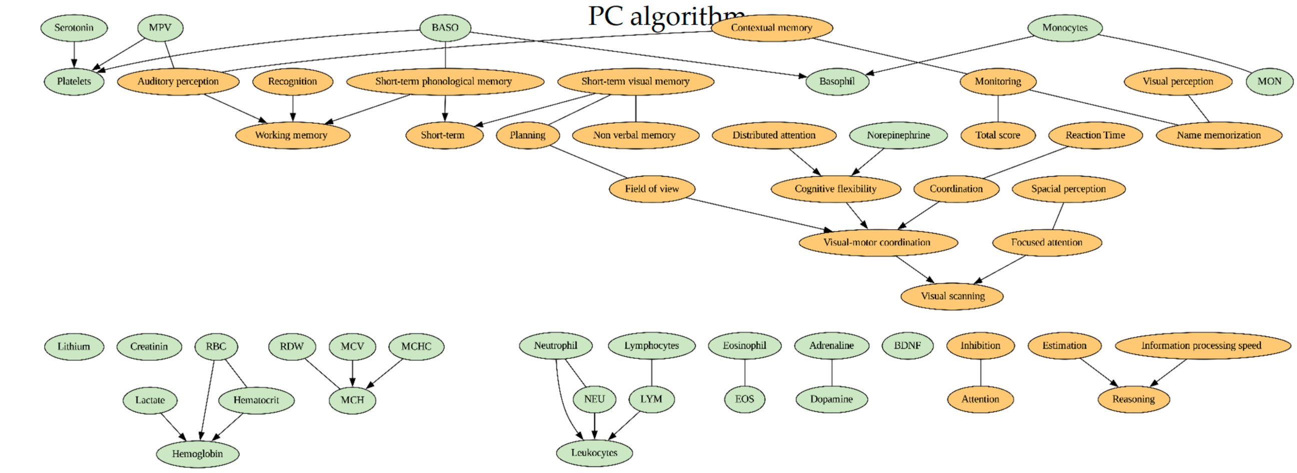

Figure S1. The result of causal analysis from PC algorithm from cognitive and biochemical parameters. Biochemical parameters - in green, cognitive parameters - in orange.

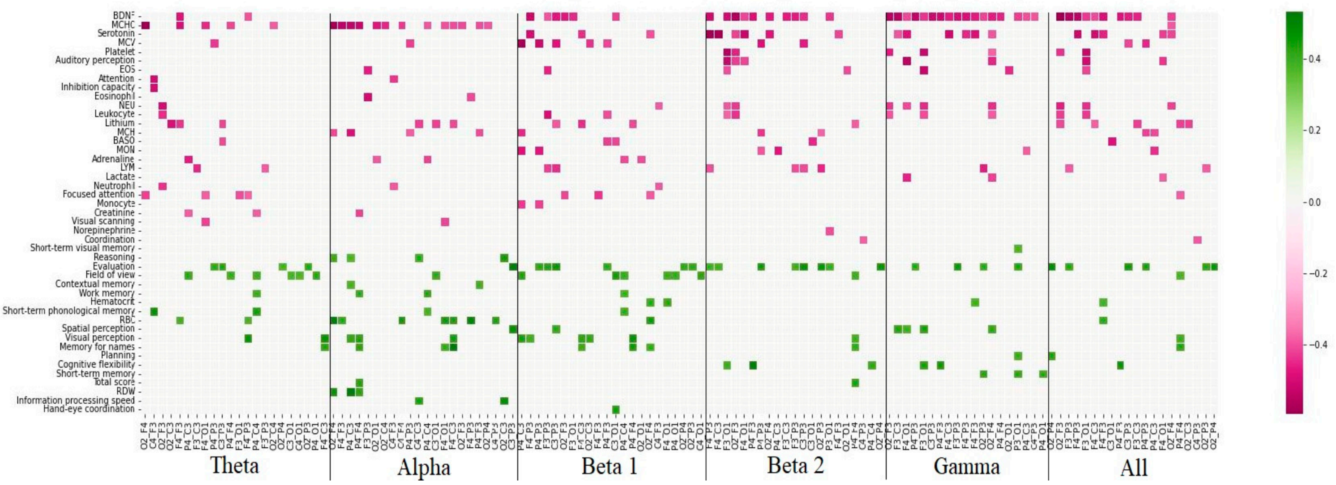

Figure S2. Spearman's rank correlation between cognitive/biochemical parameters and FC in open-eyes state before the experiment. FC calculated using the coherence method. Colors indicate the correlation coefficients.

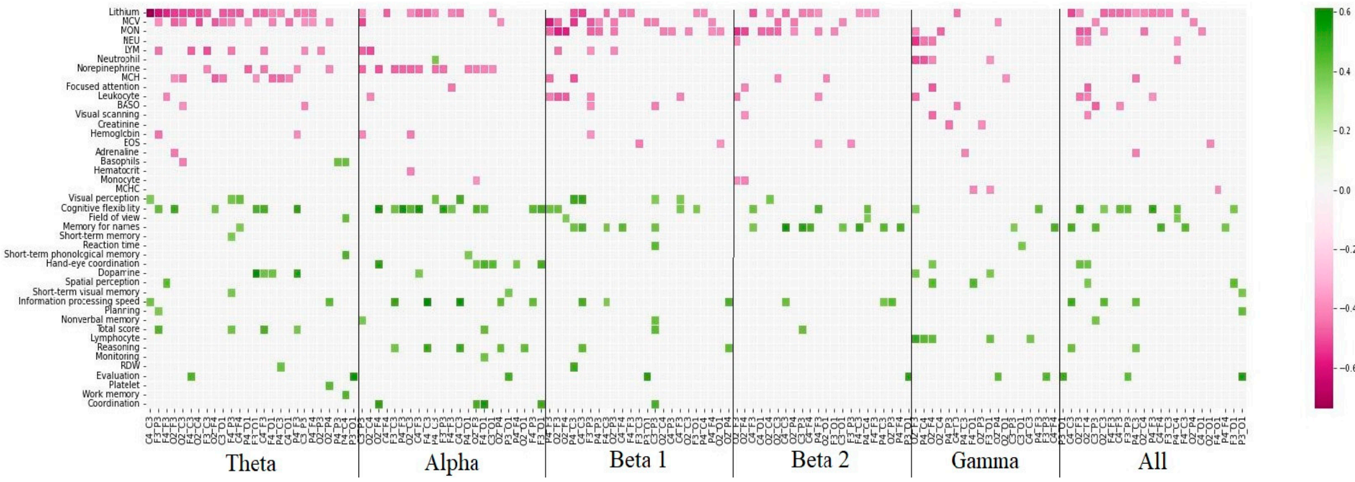

Figure S2. Spearman's rank correlation coefficient between cognitive/biochemical parameters and FC in closed-eyes state before experiment. FC calculated using the coherence method. Colors indicate the correlation coefficients.
